# X-ray and magnet to identify and remove a retained suture needle

**DOI:** 10.1016/j.jdcr.2023.10.026

**Published:** 2023-12-01

**Authors:** Jay M. Patel, Rajiv I. Nijhawan

**Affiliations:** Department of Dermatology, University of Texas Southwestern Medical Center, Dallas, Texas

**Keywords:** magnet, needle, patient safety, suture, x-ray

## Challenge

A 53-year-old male patient presented to the dermatologic surgery clinic for treatment of a 4.0 × 1.6 cm melanoma *in situ* on the right cheek. He underwent Mohs micrographic surgery with MART-1 immunostain, and the cancer was cleared in one stage. A primary repair was performed by a dermatology resident using 4-0 polyglactin-910 suture on a P-3 needle for the buried vertical mattress sutures. During placement of one of the buried vertical mattress sutures, the trainee performing the closure felt the suture needle snap. There was uncertainty among the trainee and surgical staff whether the needle broke off in the skin or was flung in the room. The trainee did not feel anything in the skin and could not find the needle anywhere in the room. The trainee then proceeded to close the remaining defect with a new suture, and only afterward alerted the attending regarding the missing needle.

## Solution

**Fig 1:**
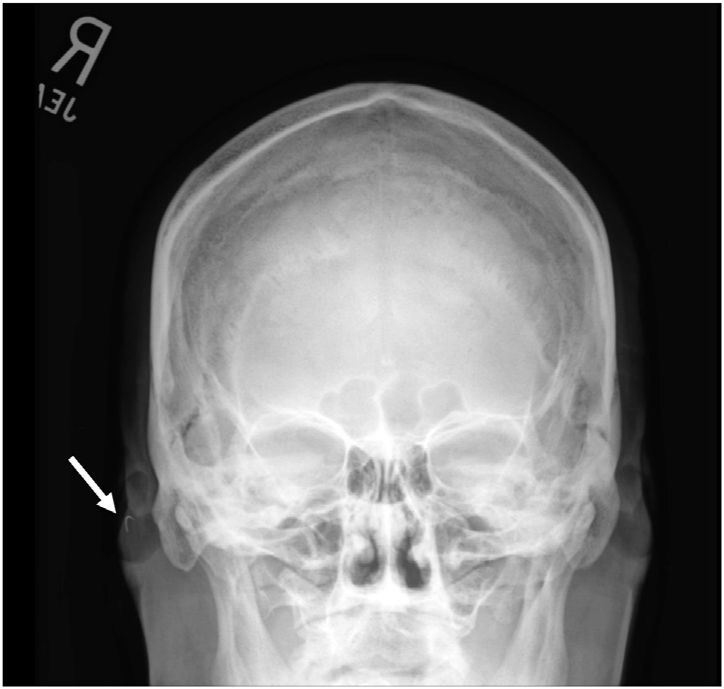
Skull x-ray indicating retained suture needle (*white arrow*).

**Fig 2:**
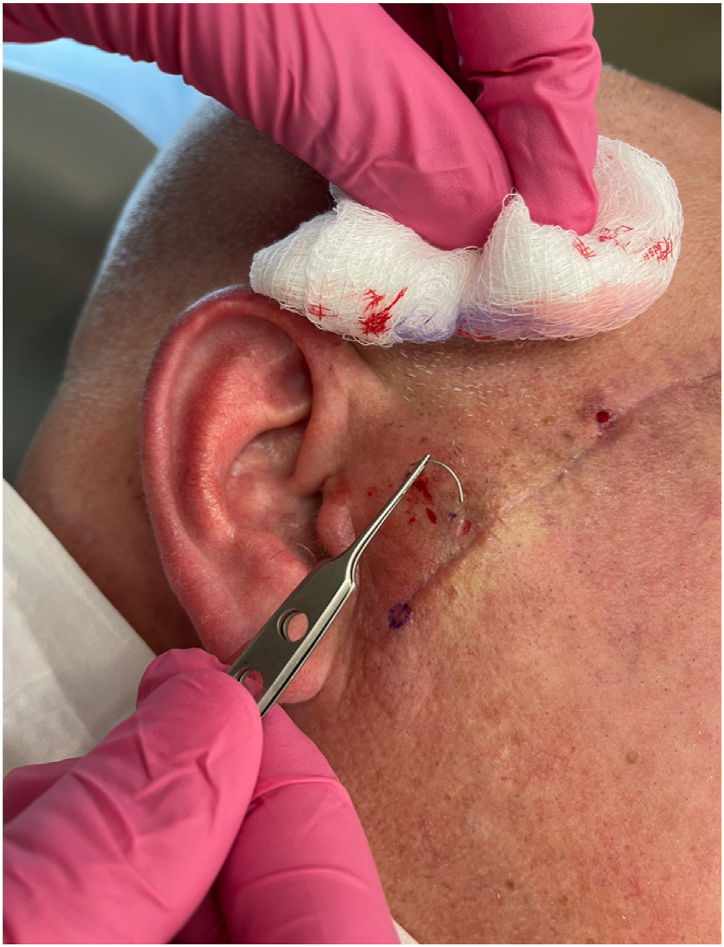
Extracted suture needle.

An x-ray of the skull was ordered to determine whether there was a retained suture needle. The x-ray demonstrated the suspected curvilinear metal needle in the right cheek (Fig 1). The patient returned for follow-up, and a magnet was used to precisely locate the needle (Video 1, available via Mendeley at https://data.mendeley.com/datasets/587n28b4bx/1). This technique requires that there are no critical structures nearby that could be damaged by the magnetic pull on the needle. The needle was subsequently easily extracted with a small incision and bishop-harmon forceps (Fig 2). This pearl highlights the utility of x-ray imaging to confirm and a magnet to identify a retained suture needle.

## Conflicts of interest

None disclosed.

